# Use of prescribed analgesics before and after exercise therapy and patient education in patients with knee or hip osteoarthritis

**DOI:** 10.1007/s00296-023-05432-0

**Published:** 2023-09-29

**Authors:** Melker S. Johansson, Anton Pottegård, Jens Søndergaard, Martin Englund, Dorte T. Grønne, Søren T. Skou, Ewa M. Roos, Jonas B. Thorlund

**Affiliations:** 1 Research Unit for Musculoskeletal Function and Physiotherapy, Department of Sports Science and Clinical Biomechanics, University of Southern Denmark, Odense, Denmark; 2 Research Unit of General Practice, Department of Public Health, University of Southern Denmark, Odense, Denmark; 3 Clinical Pharmacology, Pharmacy and Environmental Medicine, Department of Public Health, University of Southern Denmark, Odense, Denmark; 4 Clinical Epidemiology Unit, Orthopaedics, Department of Clinical Sciences Lund, Faculty of Medicine, Lund University, Lund, Sweden; 5 The Research Unit PROgrez, Department of Physiotherapy and Occupational Therapy, Næstved-Slagelse-Ringsted Hospitals, Slagelse, Denmark

**Keywords:** Osteoarthritis, Exercise therapy, Patient education, Paracetamol, Non-steroidal anti-inflammatory drugs, Opioids

## Abstract

The aim of this study was to investigate utilisation patterns of prescribed analgesics before, during, and after an exercise therapy and patient education program among patients with knee or hip osteoarthritis. This cohort study is based on data from the nationwide Good Life with osteoarthritis in Denmark (GLA:D^®^) patient-register linked with national health registries including data on prescribed analgesics. GLA:D^®^ consists of 8–12 weeks of exercise and patient education. We included 35,549 knee/hip osteoarthritis patients starting the intervention between January 2013 and November 2018. Utilisation patterns the year before, 3 months during, and the year after the intervention were investigated using total dispensed defined daily doses (DDDs) per month per 1000 population as outcome. During the year before the intervention, use of prescribed paracetamol, non-steroidal anti-inflammatory drugs (NSAIDs), and opioids increased with 85%, 79% and 22%, respectively. During the intervention, use of paracetamol decreased with 16% with a stable use the following year. Use of NSAIDs and opioids decreased with 38% and 8%, respectively, throughout the intervention and the year after. Sensitivity analyses indicated that the prescription of most analgesics changed over time. For paracetamol, NSAIDs, and opioids, 10% of analgesic users accounted for 45%, 50%, and 70%, respectively, of the total DDDs dispensed during the study period. In general, analgesic use increased the year before the intervention followed by a decrease during the intervention and the year after. A small proportion of analgesic users accounted for half or more of all paracetamol, NSAIDs, and opioids dispensed during the study period.

**Supplementary Information:**

The online version contains supplementary material available at 10.1007/s00296-023-05432-0.

## Introduction

Analgesics are traditionally used to reduce pain among patients with knee or hip osteoarthritis. However, analgesics can have negative side effects. For example, long-term paracetamol use can lead to liver damage [1–3], non-steroidal anti-inflammatory drugs (NSAIDs) increase the risk of gastrointestinal, renal, and cardiovascular events [4–6], and opioids may lead to addiction, and adverse events, such as falls and premature mortality [7–12]. It is, therefore, important to consider the risks relative to the no to small benefit of analgesics in the long-term management of osteoarthritis [1, 2, 13, 14].

Exercise therapy can reduce osteoarthritis-related pain and improve physical function [15, 16], and is a safe treatment alternative to analgesics [17, 18]. Exercise therapy for osteoarthritis-related pain has been reported to be at least as effective as paracetamol, NSAIDs, and opioids [14, 19–21], and is therefore commonly recommended as a core treatment for knee and hip osteoarthritis [13, 22]. Despite this, exercise therapy is only offered to 30–40% of patients with osteoarthritis, highlighting the suboptimal implementation of clinical guidelines [23–25]. Exercise therapy may have the potential to reduce analgesic use and associated side effects among patients already using analgesics [26]. For example, data from randomized controlled trials have indicated a 30–40% decreased risk of taking analgesics 12 months after a multimodal non-surgical treatment program including exercise therapy for patients with knee osteoarthritis [27, 28]. Also, early physical therapy (i.e., exercise and/or manual therapy within 90 days of diagnosis) has been reported to be associated with a 10% reduced opioid use among patients with knee pain [29]. Furthermore, we recently reported that the 3-months prevalence of *self-reported* analgesic use decreased from 62% before starting a standardised exercise therapy and patient education program to 44% after the intervention (i.e., at 3 months follow up) among Danish primary care patients with knee or hip osteoarthritis [26]. Among all analgesic users, 52% changed to an analgesic with a lower risk profile (e.g., from opioids to paracetamol) or stopped using analgesics after the intervention [26]. These results indicate that analgesic use may decrease after exercise therapy and patient education, but the study relied on self-reported analgesic use, which is prone to recall bias and has limited possibility to quantify changes in the amount of analgesics used. Fundamental knowledge about what types of analgesics that are used among patients with knee or hip osteoarthritis, how much, and if the use changes in relation to a treatment program in primary care (i.e., utilisation patterns) is scarce. Such knowledge is important for clinicians treating these patients, and for decision-makers developing and planning health care.

The aim of this study was to investigate utilisation patterns of prescribed analgesics before, during, and after a standardised exercise therapy and patient education program among patients with knee or hip osteoarthritis using data from a nationwide osteoarthritis patient-register linked with national register-based prescription data.

## Methods

### Data sources

We used data from the Good Life with osteoArthritis in Denmark register (GLA:D^®^; collected consecutively from inception in January 2013) [30] linked on an individual level with routinely collected health data. The GLA:D^®^ register contains patients with clinical signs of knee or hip osteoarthritis who have participated in GLA:D^®^, a standardised primary care program for knee and hip osteoarthritis. GLA:D^®^ consists of supervised neuromuscular exercises (1 h twice a week for six weeks) and group-based patient education (two 1.5-h sessions) delivered by a certified physiotherapist over 8–12 weeks. The physiotherapist supervised groups of 6–12 patients in the exercises, which were individualised to each patient’s ability (e.g., starting level, progression rate). To sustain long-term treatment effects, patients were encouraged to continue with the exercises and being physically active. The treatment program has been described in detail elsewhere [30].

We linked the GLA:D^®^ register with national health registries using the national civil registration number given to all individuals residing in Denmark. Specifically, we retrieved individual-level data on (a) dispensed prescriptions of analgesics from the Danish National Prescription Registry [31], (b) migrations and deaths from the Danish Civil Registration System, and (c) diagnostic and procedure codes from secondary health care contacts from the Danish National Patient Registry [32].

### Study population

We included patients who started the intervention (index date) from January 14, 2013, until November 30, 2018, to avoid overlap between participants’ follow-up period and the Danish COVID-19-related lockdown starting in March 2020. Study participants were required to have register-data coverage 5 years before (day − 1825 to − 1), 90 days during (day 0 to 89), and 1 year after the intervention (day 90 to 449).

We excluded patients who (a) lacked register-data coverage due to migration or death, (b) had received a cancer diagnosis within the 5 years preceding the index date (International Classification of Diseases, Tenth Revision [ICD-10] code C00-97, except C44: Other malignant neoplasms of skin), or (c) had received a substance abuse diagnosis within the year preceding the index date (ICD-10 code F11: Mental and behavioural disorders due to use of opioids).

To compare the analgesic use in the study population with the general population in Denmark, we retrieved a random general population sample matched by year of birth, sex, municipality of residence, and being alive at the time of the index date from the Danish Health Data Authority. Each study participant was matched with 20 individuals from the general population. The controls were assigned the same index date as the study participant they were matched to.

### Outcome

The outcome was total dispensed defined daily doses (DDDs) per month (i.e., 30 days) per 1000 population. We hence estimated analgesic *use* based on dispensed prescriptions.

We included oral and transdermal routes of administration of (a) paracetamol, (b) NSAIDs (divided into the following sub-groups: ibuprofen, diclofenac, etodolac, naproxen, coxibs, salicylic acid and derivatives, and other), (c) opioids (sub-groups: tramadol, codeine, oxycodone, morphine, and other), (d) gabapentinoids (sub-groups: gabapentin and pregabalin), (e) serotonin-norepinephrine reuptake inhibitors (SNRIs; sub-groups: venlafaxine and duloxetine), and (f) tricyclic antidepressants (TCAs; sub-groups: amitriptyline, nortriptyline, and imipramine). See Supplementary Table S1 for an overview of included analgesics and Anatomical Therapeutic Chemical Classification codes. Parenteral, rectal, nasal, and sublingual routes of administration were excluded (Supplementary Table S2).

### Statistical analysis

We described the study participants’ characteristics using medians with first and third quartiles and frequencies with percentages. This was done for the full study population and stratified by most symptomatic joint (knee or hip). See Supplementary Table S3 for an overview of variables used for descriptive purposes. We calculated the total number of DDDs per 1000 population for twelve 30-day intervals before the index date, three 30-day intervals during the intervention, and twelve 30-day intervals after the end of the intervention period. For combination analgesics, we calculated DDDs for each substance with an analgetic effect (see Supplementary Text S1 and Table S4 for details). This was done for each analgesic class (i.e., paracetamol, NSAIDs, opioids, gabapentinoids, SNRIs, and TCAs) and their sub-groups. Finally, to investigate any differences in utilisation patterns between patients with knee and hip osteoarthritis, we stratified the analyses by most symptomatic joint (knee or hip).

The use of opioids decreased in Denmark from 2017 and onwards following media attention focussed on tramadol use and risk of addiction, and regulatory actions targeted opioid prescribing [33]. To investigate if these temporal changes influenced our results, we conducted sensitivity analyses in which we (i) compared the total use for each analgesic class in the study population with the corresponding use in the matched general population sample, and (ii) stratified the analgesic use in the study population by calendar year (i.e., year of the index date; 2013–2015 were merged due to lower number of observations).

We described the distribution of the total use of paracetamol, NSAIDs, and opioids among analgesic users during the study period (≥ 1 dispensed prescriptions) using Lorenz curves and Gini coefficients. The Gini coefficient reflects the skewness of the Lorenz curve, where 0 reflects no skewness and 1 reflects maximal skewness. Finally, we calculated the total number of analgesic class-specific DDDs during the study period with percentages of the overall grand sum of DDDs.

We used the statistical software R (version 4.2.2, R Core Team [2023]. R: A Language and Environment for Statistical Computing. R Foundation for Statistical Computing, Vienna, Austria. https://www.R-project.org/) for all analyses.

## Results

**Fig. 1:**
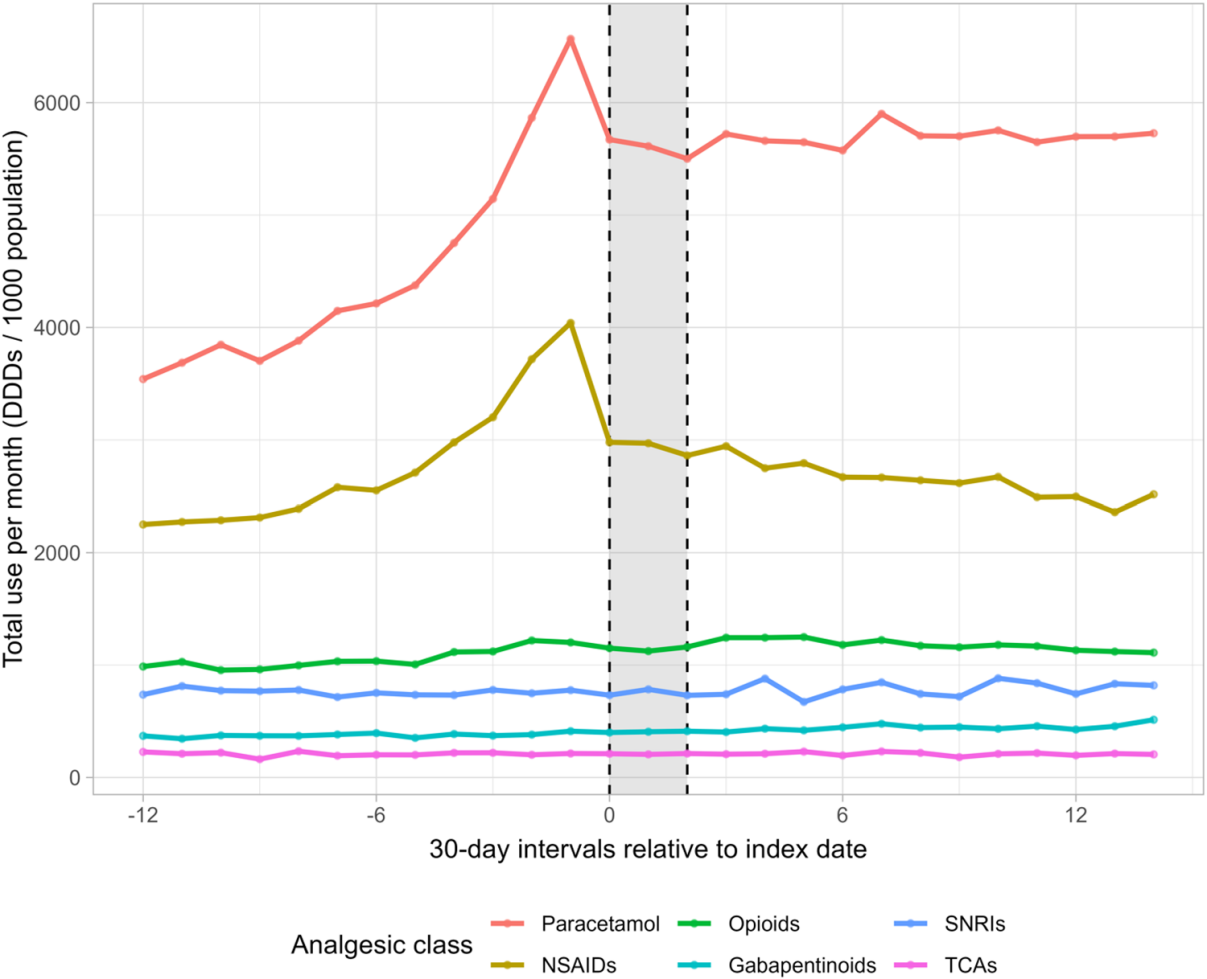
Total use of analgesics per month per 1000 population among 35,549 patients with knee or hip osteoarthritis before, during, and after an exercise therapy and patient education program in primary care in Denmark, stratified by analgesic class. The grey area reflects the intervention period. Interval 0 corresponds to the first month of the intervention. DDDs is defined daily doses, NSAIDs is non-steroidal anti-inflammatory drugs, SNRIs is serotonin-norepinephrine reuptake inhibitors, TCAs is tri-cyclic antidepressants

We included 35,549 participants in this study (Supplementary Fig. S1). The median age was 66 years, 72% were women, and the median BMI was 28 kg/m^2^ (Table 1). During the year before the intervention (i.e., pre-intervention period), the use of paracetamol and NSAIDs increased with 85% and 79%, respectively (paracetamol: from 3541 to 6567 DDDs per month per 1000 population, NSAIDs: from 2251 to 4040 DDDs per month per 1000 population; Fig. 1 and Supplementary Table S5). During the intervention period, the use of paracetamol decreased with 16% (from 6567 to 5501 DDDs per month per 1000 population). This was followed by a stable use during the year after the intervention (i.e., post-intervention period; mean: 5703 [standard deviation: 78] DDDs per month per 1000 population; Fig. 1). The use of NSAIDs decreased with 38% throughout the intervention and the year after to a level close to that of the early pre-intervention period (from 4040 to 2516 DDDs per month per 1000 population). A considerable increase in the use of opioids (22%) and gabapentinoids (39%) were observed during the pre-intervention- and whole study period, respectively (opioids: from 987 to 1202 DDDs per month per 1000 population, gabapentinoids: from 370 to 514 DDDs per month per 1000 population). The use of opioids decreased about 8% during the intervention and post-intervention period (from 1202 to 1111 DDDs per month per 1000 population). For SNRIs and TCAs, a relatively stable use pattern was observed throughout the study period (SNRIs, mean: 773 [standard deviation: 52] DDDs per month per 1000 population; TCAs, mean: 209 [standard deviation: 15] DDDs per month per 1000 population).

**Table 1:** Characteristics of Danish patients with knee or hip osteoarthritis participating in exercise therapy and patient education, overall and stratified by most symptomatic joint

Characteristic	All study participants *N* = 35,549 n (%)	Most symptomatic joint n (%)	
		Knee *n* = 26,462	Hip *n* = 9075
Age at start of intervention			
Median (Q1, Q3)	66.3 (58.9, 72.4)	65.8 (58.2, 72.0)	67.6 (61.0, 73.3)
Sex			
Women	25,544 (72)	18,938 (72)	6598 (73)
Men	10,005 (28)	7524 (28)	2477 (27)
Self-reported level of education			
Primary and lower secondary school	5544 (18)	4108 (18)	1436 (18)
General and vocational upper secondary education	3476 (11)	2626 (11)	850 (11)
Short-cycle higher education (< 3 yrs. beyond secondary school)	6187 (20)	4647 (20)	1540 (19)
Medium-cycle higher education (3–4 yrs. beyond secondary school)	12,287 (40)	9103 (40)	3182 (40)
Long cycle higher education or higher (≥ 5 yrs. beyond secondary school)	3456 (11)	2546 (11)	910 (11)
Smoking status			
Current smoker	2844 (9)	2078 (9)	766 (10)
Non-smoker	27,749 (91)	20,695 (91)	7052 (90)
Pain intensity (last month; VAS)			
Median (Q1, Q3)	48.0 (31.0, 64.0)	48.0 (31.0, 65.0)	47.0 (30.0, 64.0)
Frequency of knee/hip pain			
Never	433 (1)	345 (2)	88 (1)
Monthly	1258 (4)	985 (4)	273 (4)
Weekly	4057 (13)	3080 (13)	977 (12)
Daily	19,803 (64)	14,615 (64)	5188 (66)
Always	5338 (17)	3971 (17)	1365 (17)
BMI			
Median (Q1, Q3)	27.6 (24.6, 31.3)	28.0 (25.0, 31.8)	26.4 (23.7, 29.7)
Number of self-reported comorbidities			
None	11,216 (38)	8308 (38)	2907 (39)
1–2	15,606 (53)	11,647 (53)	3958 (53)
3 or more	2509 (9)	1858 (9)	651 (9)
Physical activity level (UCLA activity scale score)			
Low (level 1–3)	2841 (9)	2144 (9)	697 (9)
Moderate (level 4–6)	17,658 (57)	13,119 (57)	4538 (57)
High (level 7–10)	10,494 (34)	7804 (34)	2689 (34)

*N/n* number of observations, *Q1/Q3* first and third quartile, *yrs*. years, *VAS* visual analogue scale, ranging from 0–100 where 0 is ‘no pain’ and 100 is ‘worst pain imaginable’, *BMI* body mass index, number of self-reported comorbidities is based on self-reported data about the presence of 12 comorbidities, *UCLA* *activity scale* the University of California at Los Angeles Activity Rating Scale

Missing data among all study participants, *n* (%): age, 0 (0), sex, 0 (0), self-reported level of education, 4599 (13), smoking status, 4956 (14; part of the missing data is due to late introduction of the question in the baseline questionnaire), most affected joint, 12 (< 1), average pain intensity last month (VAS), 4599 (13), frequency of knee/hip pain, 4660 (13), BMI, 127 (< 1), number of self-reported comorbidities, 6218 (17; part of the missing data is due to late introduction of the questions in the baseline questionnaire), physical activity level (UCLA Activity Scale), 4556 (13)

The increase in NSAID use during the pre-intervention period was mainly attributed to an increase in the use of ibuprofen, and to a smaller extent diclofenac and naproxen (Supplementary Fig. S2 and Supplementary Table S5). The use patterns varied between the different types of opioids (Supplementary Fig. S3 and Supplementary Table S5). Tramadol use increased during the pre-intervention period followed by a U-shaped use pattern across the intervention period, and a decrease to the initial level at the end of the post-intervention-period. The use of codeine was relatively stable, while the use of oxycodone and morphine slightly increased during the study period.

**Fig. 2:**
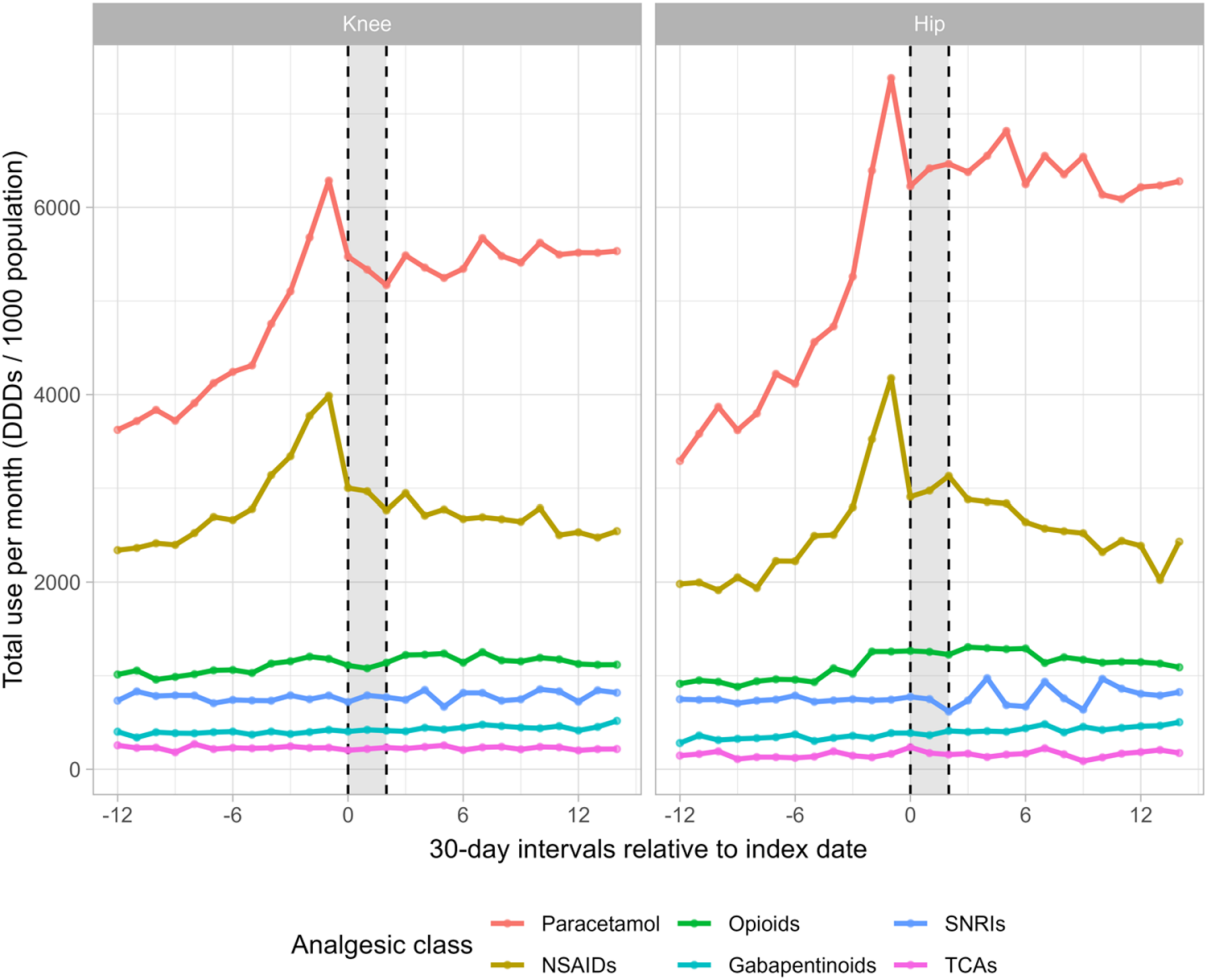
Total use of analgesics per month per 1000 population among 35,549 patients with knee or hip osteoarthritis before, during, and after an exercise therapy and patient education program in primary care in Denmark, stratified by most symptomatic joint (knee or hip). The grey area reflects the intervention period. Interval 0 corresponds to the first month of the intervention. DDDs is defined daily doses, NSAIDs is non-steroidal anti-inflammatory drugs, SNRIs is serotonin-norepinephrine reuptake inhibitors, TCAs is tri-cyclic antidepressants. Knee, n = 26,462; hip, n = 9075

When we compared the analgesic use between patients with knee and hip osteoarthritis, we found similar utilisation patterns (Fig. 2 and Supplementary Table S6). However, the relative increase in paracetamol, NSAID, and opioid use during the year before the intervention was larger among patients with hip osteoarthritis (e.g., among knee osteoarthritis patients, paracetamol, NSAID, and opioid use increased 74%, 70%, and 16%, respectively, vs. 124%, 111%, and 38% among hip osteoarthritis patients). The use of gabapentinoids increased throughout the study period in both patient groups, although to a larger extent among hip osteoarthritis patients (i.e., pre-intervention, intervention, and post-intervention period among knee osteoarthritis patients: 5%, 3%, and 27% vs. 38%, 6%, and 26% among hip osteoarthritis patients).

**Fig. 3:**
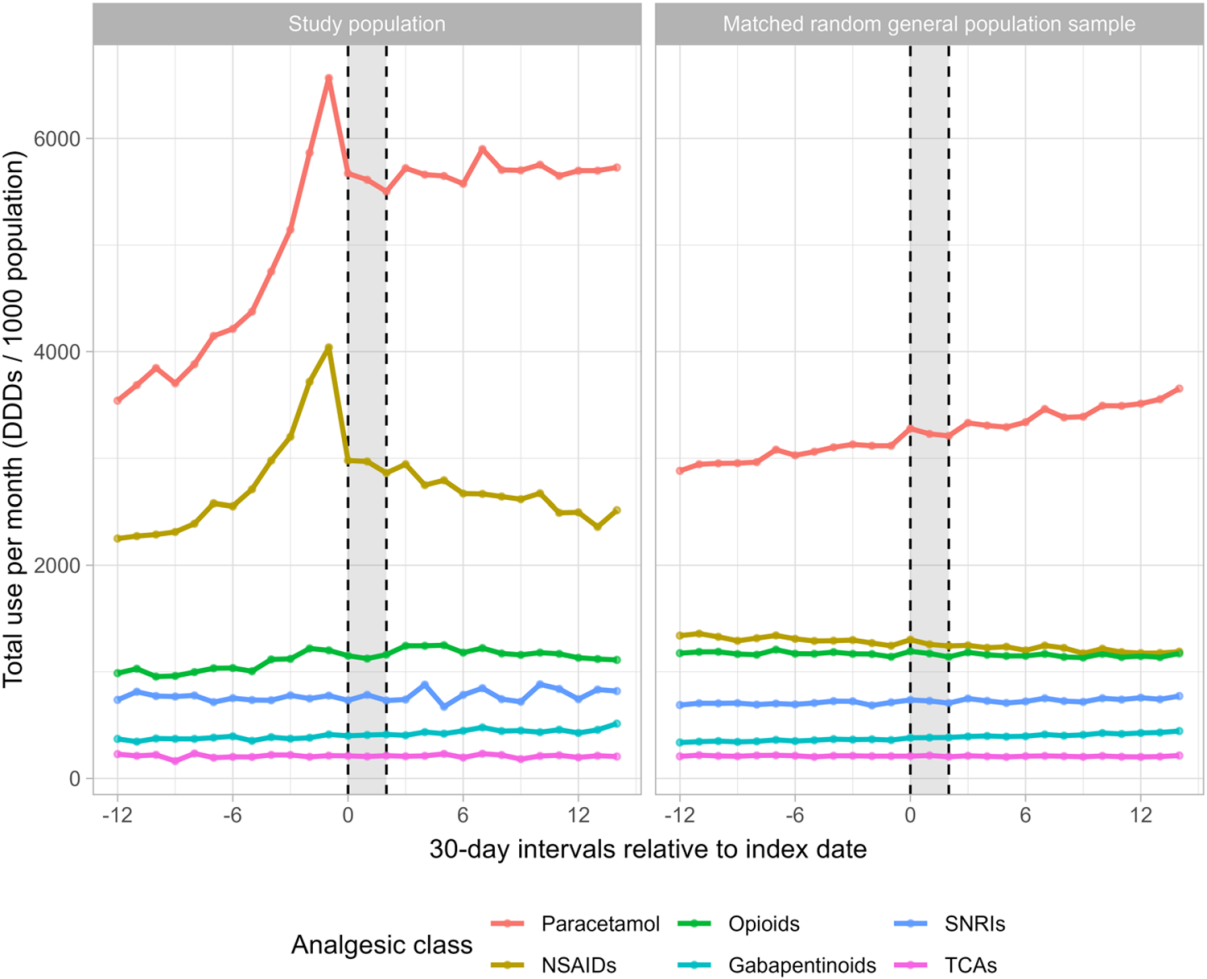
Total use of analgesics per month per 1000 population among 35,549 patients with knee or hip osteoarthritis and 675,286 matched individuals from the general population, before, during, and after an exercise therapy and patient education program in primary care in Denmark, stratified by analgesic class. The grey area reflects the intervention period. Interval 0 corresponds to the first month of the intervention. DDD is defined daily doses, NSAIDs is non-steroidal anti-inflammatory drugs, SNRIs is serotonin-norepinephrine reuptake inhibitors, TCAs is tri-cyclic antidepressants

**Fig. 4:**
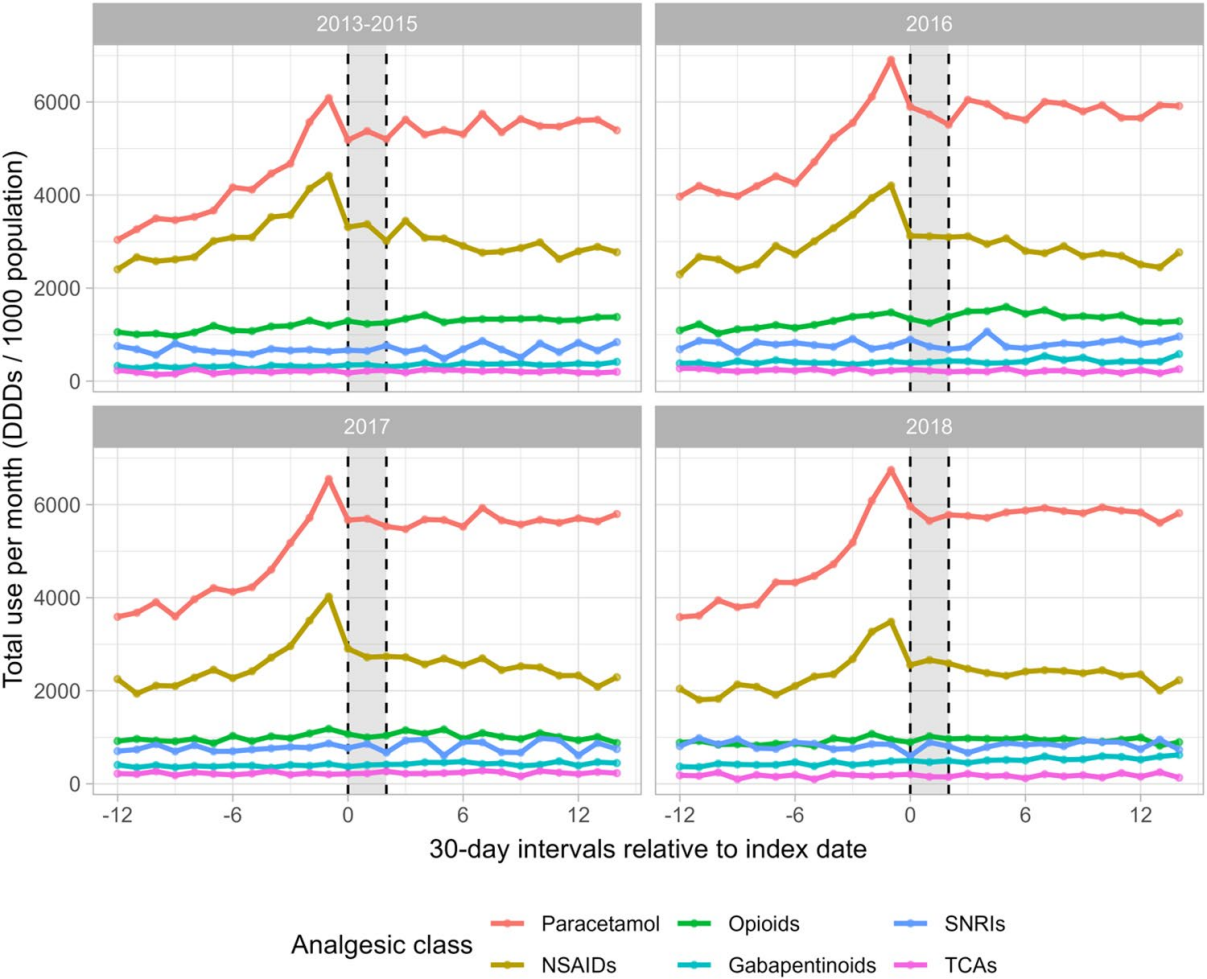
Total use of analgesics per month per 1000 population among 35,549 patients with knee or hip osteoarthritis before, during, and after an exercise therapy and patient education program in primary care in Denmark, stratified by calendar year. Interval 0 corresponds to the first month of the intervention. DDD is defined daily doses, NSAIDs is non-steroidal anti-inflammatory drugs, SNRIs is serotonin-norepinephrine reuptake inhibitors, TCAs is tri-cyclic antidepressants. 2013–2015 were collapsed due to low number of observations. 2013–2015, n = 8988; 2016, n = 8697; 2017, n = 9042; 2018, n = 8329

In the sensitivity analyses investigating the influence of temporal trends in analgesic dispensing, we observed a gradual increase (27%) in the use of paracetamol in the matched general population sample (n = 675,286) during the study period, while NSAID use decreased (11%), opioid use was stable, and the use of gabapentinoids increased (32%) (Fig. 3 and Supplementary Table S7). This indicates underlying temporal trends in the dispensing of paracetamol, NSAIDs, and gabapentinoids. When stratified by calendar year, we found temporal trends for most analgesics, although the utilisation patterns during the study period were similar between 2013–2015, 2016, 2017, and 2018 (Fig. 4 and Supplementary Table S8). Specifically, the DDDs for paracetamol and gabapentinoids increased from 2013–2015 to 2018. For opioids, the DDDs was highest during 2016, and for NSAIDs, the DDDs decreased from 2013–2015 to 2018.

**Fig. 5:**
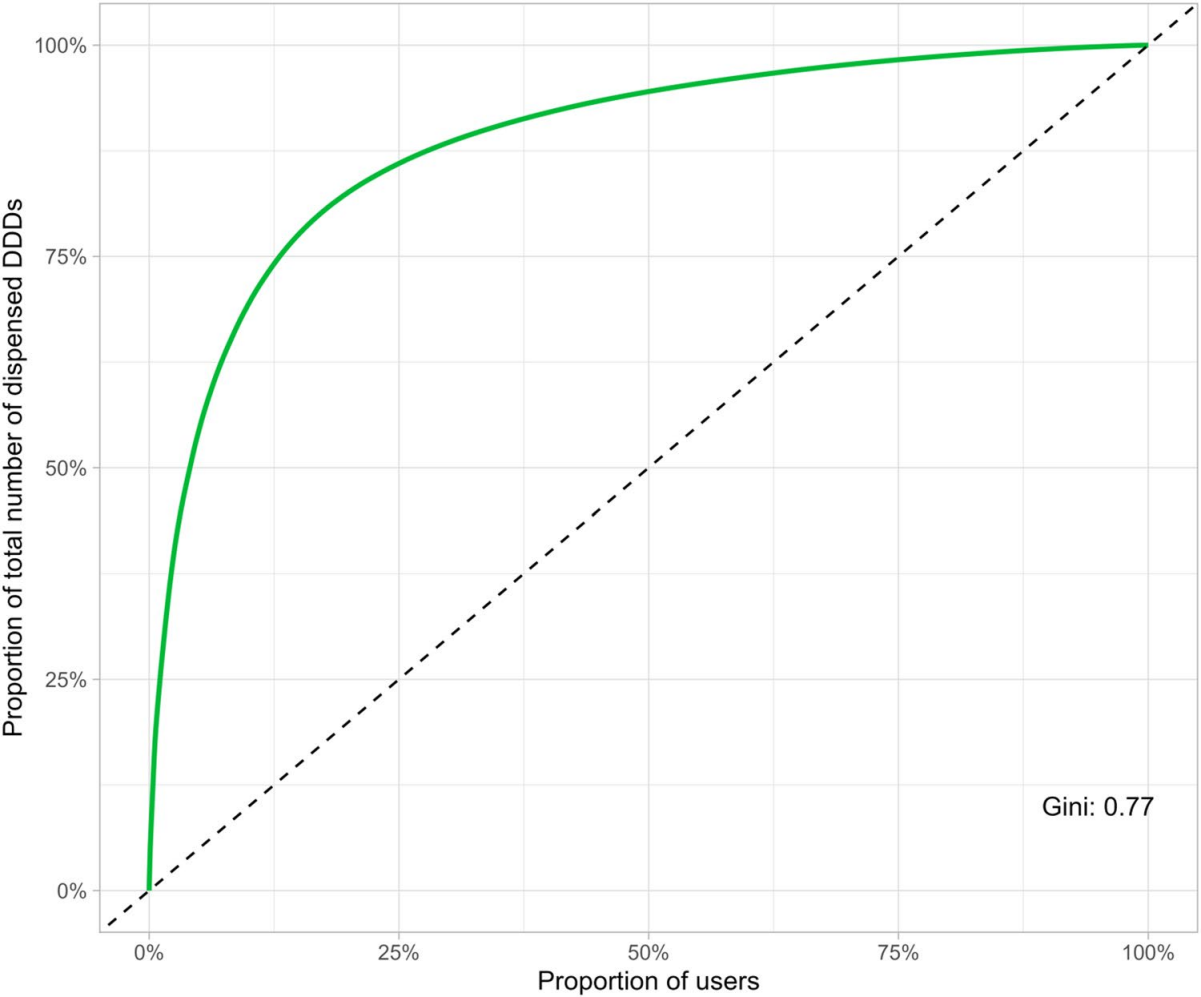
Lorenz curve illustrating the distribution of opioid use 1 year before, during, and 1 year after an exercise therapy and patient education program among 12,317 patients with knee or hip osteoarthritis in primary care in Denmark. Only patients who dispensed an opioid prescription during the study period are included. The Gini coefficient reflects the skewness of the Lorenz curve, where 0 reflects no skewness and 1 reflects maximal skewness. DDD is defined daily doses

A small proportion of all analgesic users were responsible for a large part of the total use of paracetamol, NSAIDs, and opioids dispensed during the study period, where 10% of patients using analgesic accounted for about 45%, 50%, and 70% of the total DDDs, respectively (Gini coefficients 0.53, 0.61, 0.77, respectively; Supplementary Figs. S4, S5 and Fig. 5). Of the total DDDs during the study period, dispensed prescriptions for paracetamol constituted 50%, NSAIDs 26%, opioids 11%, gabapentinoids 4%, SNRIs 7%, and TCAs 2% (Supplementary Table S9).

## Discussion

Among patients with knee or hip osteoarthritis, we found that the use of prescribed paracetamol and NSAIDs increased during the year before an exercise therapy and patient education program, followed by a decrease during the intervention and the year after. Sensitivity analyses indicated that temporal trends in the use of paracetamol, NSAIDs, opioids, and gabapentinoids likely explain a part of the observed changes in analgesic use. A small proportion of analgesic users was accountable for a large part of the total use of paracetamol, NSAIDs, and opioids.

We observed a general increase in the use of analgesics during the year before enrolment in an exercise therapy and patient education program followed by a decrease during the intervention and the year after. Since our study population is aligned by the start of the intervention, the increased use likely reflects a worsening of symptoms over time that triggered health care seeking and enrolment in GLA:D^®^. Although exercise therapy can improve osteoarthritis-related pain and physical function [15, 16], the observed decrease in analgesic use is likely due to a combination of the actual intervention, natural fluctuation of symptoms (regression to the mean), and temporal trends in prescribing patterns for analgesics. Differentiating treatment effects from the natural course and regression to the mean is difficult, in particular given the lack of a control group and temporal prescription trends occurring during the data collection period. We emphasise that this should be kept in mind when interpreting the current results. From a clinical perspective, a decreased use of analgesics and thereby a lowered risk of adverse events is of value regardless of what drives the change.

We observed similar utilisation patterns before, during, and after the intervention among knee and hip osteoarthritis patients. In line with this, comparable proportions of knee and hip osteoarthritis patients in the GLA:D^®^ register report using paracetamol, NSAIDs, and opioids, and are also similar across most baseline characteristics, such as age, sex distribution, symptom duration, pain intensity, physical function, and quality of life [34]. However, the relative increase in paracetamol, NSAID, opioid, and gabapentinoid use during the year before the intervention were larger among patients with hip osteoarthritis than knee osteoarthritis. Although speculative, potential explanations for this are differences in the effect of analgesics between knee and hip osteoarthritis patients, as the effect of NSAIDs has been reported to be lower for hip osteoarthritis patients [35], and the clinical notion that hip osteoarthritis tend to progress faster [36, 37].

In the Danish general population, most tramadol users have a sporadic or short-term use while few have a heavy or long-term use [33]. However, long-term opioid use is about four times more prevalent among individuals with knee or hip osteoarthritis than in the general population [38–41], and the distribution of analgesic use could therefore be different in an osteoarthritis population. In agreement with this, we found a skewed distribution of opioid use where 10% of all opioid users accounted for 70% of all opioids dispensed during the study period (Gini coefficient: 0.77). In comparison, 10% of tramadol users in the Danish general population used about 50% of all dispensed tramadol in 2019 (Gini coefficient: 0.70) [33]. Furthermore, the use of paracetamol, NSAIDs, and opioids was generally higher in the study population compared to the Danish general population [33, 42, 43]. The high use of analgesics is **not** in agreement with the most recent international clinical guidelines for knee and hip osteoarthritis [13, 22]. To reduce the total use of analgesics and, thereby, the risk of adverse events among patients with knee or hip osteoarthritis, one potential strategy is to target ‘heavy’ or long-term users of paracetamol, NSAIDs, or opioids in deprescribing interventions and regulatory actions. However, the role of exercise therapy and patient education in deprescribing strategies requires further investigation.

### Strengths and limitations

Paracetamol, NSAIDs, and codeine combination drugs sold over the counter and analgesics used during hospital admissions are not included in our analyses. In Denmark, 22% of the total paracetamol and NSAID sales, respectively, are sold over the counter [42, 43]. However, over the counter analgesics have sales restrictions (e.g., small package sizes) and are not reimbursed, which makes individuals with chronic pain conditions such as osteoarthritis more likely to rely on prescription-based analgesics [31, 43]. Therefore, the potential influence of not including over the counter analgesics on our results is likely small. Common to all studies using register-based prescription data, we do not know whether the dispensed analgesics were consumed and if so when it was consumed. Also, we do not know the specific pain indication for the prescribed analgesics. However, all study participants had signed up for a standardised primary care program for patients with knee or hip osteoarthritis, which suggests the knee or hip pain to be their main pain complaint. The main indication for SNRIs and TCAs is depression and not pain. Since the prevalence of depression was 4% (based on ICD-10 codes F32-33 within the year before the intervention, or self-reported in the GLA:D^®^ baseline questionnaire), we judged the potential influence of this on the results to be low and did not investigate this further. Since we used data from a large nationwide patient database linked with national prescription registry data covering all dispensed prescriptions in Denmark, we consider the generalizability of these results to similar patient populations to be good.

## Conclusions

Among patients with knee or hip osteoarthritis, the use of several analgesics increased during the year before an exercise therapy and patient education program, followed by a decrease during the intervention and the year after. The observed changes in analgesic use are likely due to a combination of the actual intervention, natural fluctuation of symptoms (including regression to the mean), and temporal trends in prescribing patterns for analgesics. A small proportion of analgesic users accounted for half or more of all paracetamol, NSAIDs, and opioids dispensed during the study period. Deprescribing initiatives and regulatory actions may be needed to reduce the high use of paracetamol, NSAIDs, and opioids and thereby the risk of adverse events among Danish patients with osteoarthritis. The role of exercise therapy and patient education in deprescribing requires further investigation.

### Supplementary Information

Below is the link to the electronic supplementary material.

Supplementary file1 (PDF 1149 kb)

## Data Availability

The data underlying this article cannot be shared publicly due to potentially identifiable or sensitive information (General Data Protection Regulation, European Union). Data may be accessed by contacting GLA:D^®^ (https://gladinternational.org/contact/).
