# Supplementary material for: Use of prescribed analgesics before and after exercise therapy and patient education in patients with knee or hip osteoarthritis
Source: Rheumatol Int. 2023 Sep 29;44(2):319–28. doi: 10.1007/s00296-023-05432-0 (PMC10796603; doi:10.1007/s00296-023-05432-0)
Supplement: Supplementary file 1 — Supplementary file1 (PDF 1149 kb) [file 296_2023_5432_MOESM1_ESM.pdf]

SUPPLEMENTARY FILES

Use of prescribed analgesics before and after exercise therapy and patient education in patients with knee or hip osteoarthritis

Authors:

Melker S. Johansson[1,2], Anton Pottegård[3], Jens Søndergaard[2], Martin Englund[4], Dorte T. Grønne[1], Søren T. Skou[1,5], Ewa M. Roos[1], and Jonas B. Thorlund[1,2]

Affiliations:

[1] Research Unit for Musculoskeletal Function and Physiotherapy, Department of Sports Science and Clinical Biomechanics, University of Southern Denmark, Odense, Denmark

[2] Research Unit of General Practice, Department of Public Health, University of Southern Denmark, Odense, Denmark

[3] Clinical Pharmacology, Pharmacy and Environmental Medicine; Department of Public Health, University of Southern Denmark; Odense; Denmark

[4] Clinical Epidemiology Unit, Orthopedics, Department of Clinical Sciences Lund, Faculty of Medicine, Lund University, Lund, Sweden

[5] The Research Unit PROgrez, Department of Physiotherapy and Occupational Therapy, Næstved-Slagelse-Ringsted Hospitals, Slagelse, Denmark

LIST OF CONTENT

METHODS 3

OVERVIEW OF INCLUDED ATC-CODES 3

OVERVIEW OF EXCLUDED ATC-CODES AND DOSE FORMULATIONS 5

DEFINITION OF VARIABLES 6

TEXT S1: CALCULATION OF DDDS FOR COMBINATION ANALGESIC PRODUCTS 8

RESULTS 10

FORMATION OF STUDY POPULATION 10

UTILIZATION PATTERNS 11

LORENZ CURVES 17

TOTAL DDDs DISPENSED DURING STUDY PERIOD 19

METHODS

OVERVIEW OF INCLUDED ATC-CODES

Table S1. Overview of ATC-codes, analgesic class, routes of administration, and mg/DDD of included analgesics

ATC	Drug	Analgesic group, sub-group	Adm. R	mg/DDD
		**PARACETAMOL**		
N02BE01	Paracetamol	Paracetamol	O	3000.0
		**NSAIDs**		
M01AA01	Phenylbutazone	NSAIDs, other	O	300
M01AB01	Indometacin	NSAIDs, other	O	100
M01AB02	Sulindac	NSAIDs, other	O	400
M01AB03	Tolmetin	NSAIDs, other	O	700
M01AB05	Diclofenac	NSAIDs, diclofenac	O	100
M01AB55	Diclofenac, combinations	NSAIDs, diclofenac	O	100
M01AB08	Etodolac	NSAIDs, etodolac	O	400
M01AB16	Aceclofenac	NSAIDs, other	O	200
M01AC01	Piroxicam	NSAIDs, other	O	20
M01AC02	Tenoxicam	NSAIDs, other	O	20
M01AC05	Lornoxicam	NSAIDs, other	O	12
M01AC06	Meloxicam	NSAIDs, other	O	15
M01AE01	Ibuprofen	NSAIDs, ibuprofen	O	1200
M01AE02	Naproxen	NSAIDs, naproxen	O	500
M01AE03	Ketoprofen	NSAIDs, other	O	150
M01AE04	Fenoprofen	NSAIDs, other	O	1200
M01AE09	Flurbiprofen	NSAIDs, other	O	200
M01AE11	Tiaprofenic acid	NSAIDs, other	O	600
M01AE14	Dexibuprofen	NSAIDs, other	O	800
M01AE17	Dexketoprofen	NSAIDs, other	O	75
M01AE52	Naproxen and esomeprazole	NSAIDs, naproxen	O	500
M01AG02	Tolfenamic acid	NSAIDs, other	O	300
M01AH01	Celecoxib	NSAIDs, coxibs	O	200
M01AH02	Rofecoxib	NSAIDs, coxibs	O	25
M01AH05	Etoricoxib	NSAIDs, coxibs	O	60
N02BA01	Acetylsalicylic acid (Aspirin, Idotyl, Magnyl)	NSAIDs, salicylic acid and derivatives	O	3000.0
N02BA11	Diflunisal (Donobid)	NSAIDs, salicylic acid and derivatives	O	750.0
N02BA51	Acetylsalicylic acid, caffeine (500 + 50 mg, Migpriv, Treo, Triplo)	NSAIDs, salicylic acid and derivatives	O	3000.0
		**OPIOIDS**		
N02AA01	Morphine	Opioid, morphine	O	100.0
N02AA03	Hydromorphone	Opioid, other	O	20.0
N02AA04	Nicomorphine	Opioid, other	O	30.0
N02AA05	Oxycodone	Opioid, oxycodone	O	75.0
N02AA55	Oxycodone and naloxone	Opioid, oxycodone	O	75.0
N02AB01	Ketobemidone	Opioid, other	O	50.0
N02AB02	Pethidine	Opioid, other	O	400.0
N02AB03	Fentanyl	Opioid, other	TD	1.2
N02AC04	Dextropropoxyphene	Opioid, other	O	200.0/300.0[a]
N02AD01	Pentazocine	Opioid, other	O	200.0
N02AE01	Buprenorphine	Opioid, other	TD	1.2
N02AG02	Ketobemidone, antispasmodics	Opioid, other	O	25.0[b]

**Table S1**. Continued.				
**ATC**	**Drug**	**Analgesic group, sub-group**	**Adm. R**	**mg/DDD**
N02AJ06	Codeine, paracetamol (30 + 500 mg; Pinex Comp/Citodon)	Opioid, codeine	O	90.0[c]
N02AJ06	Codeine, paracetamol (30.6 + 500 mg; Kodipar)	Opioid, codeine	O	91.8[c]
N02AJ06	Codeine, paracetamol (28.4 + 400 mg; Fortamol)	Opioid, codeine	O	114.8[d]
N02AJ07	Codeine, acetylsalicylic acid (9.6 + 500 mg; Kodimagnyl)	Opioid, codeine	O	57.6[e]
N02AJ07	Codeine, acetylsalicylic acid (10 + 500 mg; Codyl)	Opioid, codeine	O	60.0[e]
N02BA75	Codeine, caffeine, propyphenazone, salicylamide, magnesium oxide (9.6 + 50 + 150 + 250 + 80 mg; Kodamid)	Opioid, codeine	O	57.6[e]
R05DA04	Codeine	Opioid, codeine	O	100.0
N02AX02	Tramadol	Opioid, tramadol	O	300.0
N02AX06	Tapentadol	Opioid, other	O	400.0
		**GABAPENTINOIDS**		
N03AX12	Gabapentin	Gabapentinoids, gabapentin	O	1800.0
N03AX16	Pregabalin	Gabapentinoids, pregabalin	O	300.0
		**SNRIs**		
N06AX16	Venlafaxine	SNRIs, venlafaxine	O	100.0
N06AX21	Duloxetine	SNRIs, duloxetine	O	?
		**TCAs**		
N06AA02	Imipramine	TCAs, imipramine	O	100.0
N06AA09	Amitriptyline	TCAs, amitriptyline	O	75.0
N06AA10	Nortriptyline	TCAs, nortriptyline	O	75.0

ATC, Anatomical Therapeutical Chemical code
DDD, defined daily dose
Adm. R, route of administration
NSAIDs, non-steroidal anti-inflammatory drug
SNRIs, serotonin–norepinephrine reuptake inhibitors
TCAs, tricyclic antidepressants

OVERVIEW OF EXCLUDED ATC-CODES AND DOSE FORMULATIONS

Table S2. Overview of excluded ATC-codes and routes of administration

ATC	Drug	Adm. R
M01AX*	Other anti-inflammatory and antirheumatic agents, non-steroids (e.g., glucosamine)	
-	-	P
-	-	R
-	-	N
-	-	SL

ATC-codes, Anatomical Therapeutical Chemical Classification codes
Adm. R, route of administration
P, parenteral
R, rectal
N, nasal
SL, sublingual
O, oral

DEFINITION OF VARIABLES

**Table S3.** Overview of variables used for descriptive purposes	
**Variable**	**Possible responses and modifications/derivation**
Sex, categorical	Female<br>Male<br>Derived from study participants' CPR-number.
Age (years), continuous	Corresponds to age at start of the GLA:D® program.<br>Derived from study participants' CPR-number and date of entering GLA:D®.
Self-reported level of education, categorical<br>*'What is the highest level of education that you have completed?'*	Primary and lower secondary school<br>General and vocational upper secondary education<br>Short-cycle higher education, <3 yrs. beyond secondary school<br>Medium-cycle higher education, 3-4 yrs. beyond secondary school<br>Long cycle higher education or higher, ≥5 yrs. beyond secondary school
Smoking status, categorical<br>*'Do you currently smoke?'*	Yes<br>No
Most affected joint	Knee<br>Hip
Average pain intensity last month (baseline), continuous<br>'On a scale from 'no pain' (0) to 'worst pain imaginable' (100), please mark the number that best represents your average knee/hip pain intensity during the last month.'	Measured on a 100 mm VAS.<br>0 no pain, 100 worst imaginable pain.
Frequency of knee/hip pain	Never<br>Monthly<br>Weekly<br>Daily<br>Always
BMI (kg/m^2), continuous	Calculated from weight in kilograms divided by height in meters squared.
Number of self-reported comorbidities, continuous	Based on self-reported data about the presence of hypertension, heart disease, stomach ulcer or gastrointestinal disease, respiratory disease, diabetes, kidney- or liver disease, anaemia, cancer, depression, rheumatoid arthritis, neurological disease, and other medical disease (i.e., maximum number of comorbidities was 12).

**Table S3**. Continued	
**Variable**	**Possible responses and modifications/derivation**
Physical activity level, UCLA Activity score, categorical<br>*'What is your current activity level? Consider your activity level during the last 4 weeks. Level 10 is very high and 1 is very low.'*	Responses:<br>10/9: Regularly/Sometimes participates in impact sports such as jogging, soccer, handball, badminton, tennis, skiing, heavy labor, or backpacking.<br>8/7: Regularly/Sometimes participates in active events, such as bicycling for a long time, golf, or hard gymnastics/fitness.<br>6/5: Regularly/Sometimes participates in moderate activities such as swimming, bicycling, a long walk, or could do unlimited housework or shopping.<br>4/3: Regularly/Sometimes participates in mild activities such as walking, limited housework, and limited shopping.<br>2: Mostly inactive or restricted to minimum activities of daily living.<br>1: Wholly inactive, dependent on others, and cannot leave residence.<br>Dichotomized into:<br>Response 1-6: Low-to-moderate physical activity level<br>Response ≥7: High physical activity level
CPR-number, Central Person Register-number<br>GLA:D®, Good Life with osteoArthritis in Denmark<br>yrs., years<br>VAS, visual analogue scale<br>BMI, body mass index<br>UCLA Activity Score, the University of California at Los Angeles Activity Rating Scale	

TEXT S1: CALCULATION OF DDDS FOR COMBINATION ANALGESIC

PRODUCTS

The number of DDDs of a dispensed prescription for the combination analgesics N02AJ06, N02AJ07, or N02BA75 were split and calculated for each relevant substance using the following approach:

$$n\ DDD_{comb.\ analgesic} = n\ DDD_{substance\ x} + n\ DDD_{substance\ y}, where$$

$$n\ DDD_{substance\ x} = \frac{\left(n\ tbl/DDD_{comb.\ analgesic} \times mg_x/tbl\right)}{mg_x/DDD_x} \times n\ DDDs_{dispensed\ prescription}$$

$$n\ DDD_{substance\ x} = \frac{\left(mg_x/DDD_{comb.\ analgesic}\right)}{mg_x/DDD_x} \times n\ DDDs_{dispensed\ prescription}, and$$

$$n\ DDD_{substance\ y} = \frac{\left(mg_y/DDD_{comb.\ analgesic}\right)}{mg_y/DDD_y} \times n\ DDDs_{dispensed\ prescription},$$

$$where\ n\ DDDs_{dispensed\ prescription} = \frac{(n\ tbl \times mg/tbl)}{n\ tbl/DDD \times mg/tbl} = \frac{mg/dispensed\ prescription}{n\ DDD_{comb.\ analgesic}}$$

Example

1 package Kodipar (30.6 mg codeine + 500 mg paracetamol) including 20 tablets:

$$DDD_{Kodipar} = DDD_{codeine} + DDD_{paracetamol}$$

$$DDD_{codeine} = \frac{\left(3\ tbl/DDD_{Kodipar} \times 30.6\ mg/tbl\right)}{100\ mg/DDD_{codeine}} \times 6.667\ DDDs_{dispensed\ prescription}$$

$$\left\{where\ 6.667\ DDDs_{dispensed\ prescription} = \frac{(20\ tbl \times 30.6\ mg/tbl)}{91.795}\right\}$$

$$\boldsymbol{DDD_{codeine} = 0.918 \times 6.667 = 6.120}$$

$$DDD_{paracetamol} = \frac{\left(3\ tbl/DDD_{Kodipar} \times 500\ mg/tbl\right)}{3000\ mg/DDD_{paracetamol}} \times 6.667\ DDDs_{dispensed\ prescription}$$

$$\boldsymbol{DDD_{paracetamol} = 0.500 \times 6.667 = 3.334}$$

$$DDD_{Kodipar} = 6.120\ DDD_{codeine} + 3.334\ DDD_{paracetamol} = 9{,}454\ DDD_{Kodipar}$$

Table S4. Overview of combination drugs where the number of DDDs have been calculated for each active substance

ATC	Drug	Analgesic class, sub-group	N tbl/DDD[a]	mg/DDD
N02AJ06	Codeine, paracetamol (Pinex Comp/Citodon; 30 + 500 mg)	Opioid, codeine	3	90.0
N02AJ06	Codeine, paracetamol (Pinex Comp/Citodon; 30 + 500 mg)	Paracetamol	3	1500.0
N02AJ06	Codeine, paracetamol (Kodipar; 30.6 + 500 mg)	Opioid, codeine	3	91.8
N02AJ06	Codeine, paracetamol (Kodipar; 30.6 + 500 mg)	Paracetamol	3	1500.0
N02AJ06	Codeine, paracetamol (Fortamol; 28.4 + 400 mg)	Opioid, codeine	4	114.8
N02AJ06	Codeine, paracetamol (Fortamol; 28.4 + 400 mg)	Paracetamol	4	1600.0
N02AJ07	Codeine, acetylsalicylic acid (Kodimagnyl; 9.6 + 500 mg)	Opioid, codeine	6	57.6
N02AJ07	Codeine, acetylsalicylic acid (Kodimagnyl; 9.6 + 500 mg)	NSAIDs, salicylic acid and derivatives	6	3000.0
N02AJ07	Codeine, acetylsalicylic acid (Codyl; 10 + 500 mg)	Opioid, codeine	6	60.0
N02AJ07	Codeine, acetylsalicylic acid (Codyl; 10 + 500 mg)	NSAIDs, salicylic acid and derivatives	6	3000.0
N02BA75	Codeine, salicylamide, caffeine, propyphenazone, magnesium oxide (Kodamid; 9.6 + 250 + 50 + 150 + 80 mg)	Opioid, codeine	6	57.6
N02BA75	Codeine, salicylamide, caffeine, propyphenazone, magnesium oxide (Kodamid; 9.6 + 250 + 50 + 150 + 80 mg)	NSAIDs, salicylic acid and derivatives	6	57.6

DDDs, defined daily doses
tbl, tablets
NSAIDs, non-steroidal anti-inflammatory drugs
[a] As defined by the World Health Organization Collaborating Centre for Drug Statistics Methodology (https://www.whocc.no/ddd/list_of_ddds_combined_products/).

RESULTS

FORMATION OF STUDY POPULATION

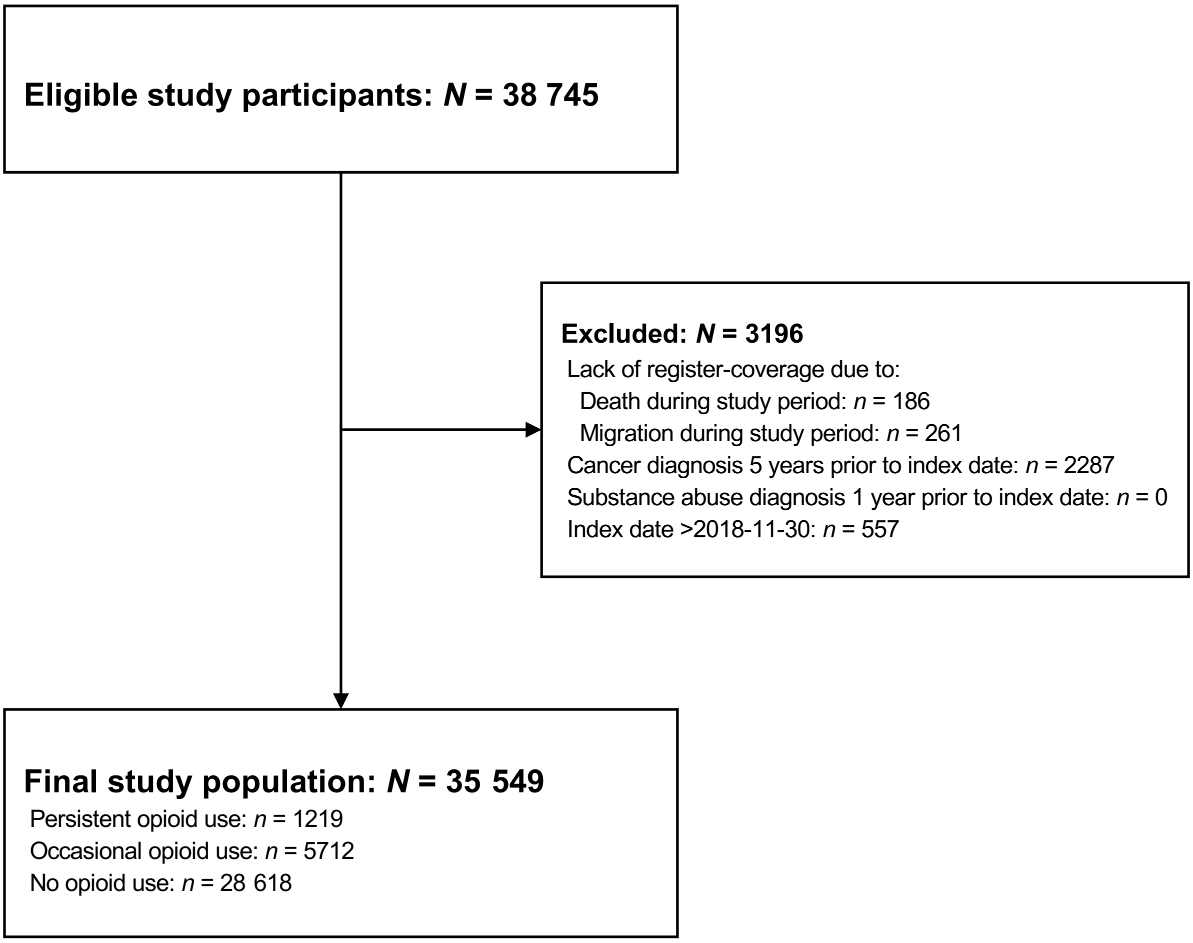

Figure S1. Formation of study population. The sum across reasons for exclusion exceeds 3196 since some study participants fulfilled more than one exclusion criterion.

Table S5. Overall, analgesic class-, and analgesic sub-group specific number of DDDs among 35 549 individuals with knee or hip osteoarthritis participating in a supervised exercise and patient education program in primary care in Denmark

Analgesic class / sub-group	30-day interval relative to index date Number of DDDs per 1000 population																										
	Pre-intervention period intervals												Intervention period intervals			Post-intervention period intervals											
	**-12**	**-11**	**-10**	**-9**	**-8**	**-7**	**-6**	**-5**	**-4**	**-3**	**-2**	**-1**	**0**	**1**	**2**	**3**	**4**	**5**	**6**	**7**	**8**	**9**	**10**	**11**	**12**	**13**	**14**
Overall	8114	8358	8458	8279	8652	9052	9151	9379	10184	10838	12136	13212	11145	11104	10880	11262	11178	11013	10849	11345	10925	10825	11131	10822	10693	10681	10894
Paracetamol	3541	3686	3846	3703	3882	4148	4214	4375	4751	5144	5866	6567	5672	5613	5501	5721	5660	5648	5575	5901	5705	5701	5754	5649	5698	5699	5728
NSAIDs	2251	2273	2288	2312	2390	2579	2551	2709	2978	3202	3718	4040	2979	2970	2861	2944	2748	2793	2669	2665	2641	2616	2671	2491	2496	2360	2516
Ibuprofen	1598	1632	1680	1646	1738	1864	1905	1950	2248	2397	2799	3142	2229	2201	2110	2200	2053	2131	2002	2020	2033	1997	2009	1919	1903	1754	1920
Diclofenac	180	198	178	221	183	208	206	185	233	242	265	275	206	190	211	229	216	184	183	175	148	160	198	168	131	175	151
Etodolac	113	126	110	105	111	125	108	111	85	123	144	124	100	122	116	92	97	89	91	107	97	82	99	80	69	85	82
Naproxen	199	187	187	214	209	235	219	297	268	292	337	347	307	302	285	281	234	251	265	251	236	252	246	193	266	236	236
Coxibs	32	4	23	11	23	20	6	24	4	9	21	14	9	8	15	16	28	13	23	12	10	17	23	16	17	22	14
Salicylic acid derivatives	23	25	27	19	24	29	24	25	27	28	25	25	26	24	28	23	21	22	23	22	22	21	20	25	19	24	21
Other	106	101	85	96	101	99	82	117	113	112	125	113	102	122	95	104	99	103	81	79	94	86	77	90	92	64	92
Opioids	987	1029	955	962	997	1034	1036	1006	1116	1121	1219	1202	1151	1124	1161	1244	1244	1250	1180	1223	1172	1159	1180	1169	1132	1120	1111
Tramadol	557	537	524	524	526	567	579	544	615	610	697	685	636	613	627	693	676	668	606	629	601	590	620	593	598	531	553
Codeine	296	323	288	283	311	327	299	308	341	351	360	366	360	332	337	331	344	336	344	335	324	331	325	319	310	339	290
Oxycodone	45	48	45	46	46	49	45	45	58	47	48	40	48	56	65	80	92	104	109	110	109	102	112	113	96	110	113
Morphine	59	84	66	72	81	64	76	81	68	78	77	81	75	90	96	96	92	109	89	102	110	96	86	107	98	88	107
Other	30	36	31	37	33	27	36	28	34	35	36	30	32	33	36	45	41	31	32	47	29	40	38	37	31	52	47
Gabapentinoids	370	345	374	371	371	383	395	353	387	372	381	412	400	407	412	404	435	420	446	478	444	449	433	457	426	456	514
Gabapentin	160	164	158	175	170	179	178	167	193	188	191	200	205	199	202	216	228	205	241	248	235	236	235	253	229	264	286
Pregabalin	211	181	216	196	200	204	217	186	194	184	190	213	195	208	210	189	206	214	205	230	209	213	199	204	198	191	228
SNRIs	737	813	773	768	779	716	753	735	733	779	749	777	732	784	731	740	880	672	784	847	744	719	883	840	744	834	820
Venlafaxine	540	599	535	590	576	484	561	544	541	564	531	561	516	563	533	555	640	440	594	604	515	505	631	614	486	585	580
Duloxetine	197	214	238	179	203	232	192	191	192	215	219	216	217	221	198	186	240	232	190	242	229	214	251	226	257	248	239
TCAs	228	212	221	163	234	194	202	201	219	220	202	214	212	206	213	208	211	230	196	232	219	181	210	217	197	213	206
Amitriptyline	87	82	79	66	96	73	69	78	75	91	77	80	82	73	90	77	95	98	65	92	76	76	94	85	72	88	76
Nortriptyline	127	119	126	89	126	107	120	115	133	115	111	124	115	120	114	114	107	119	119	126	129	97	103	119	114	113	115
Imipramine	14	11	16	8	12	14	13	8	12	14	14	10	15	12	10	17	10	13	11	14	14	8	14	13	11	12	15

DDD, defined daily dose
NSAIDs, non-steroidal anti-inflammatory drugs
SNRIs, serotonin-norepinephrine reuptake inhibitors
TCAs, tricyclic antidepressants
30-day interval ‘0’ corresponds to the first 30-days of the intervention.

Table S6. Analgesic class-specific number of DDDs among 35 549 individuals with knee or hip osteoarthritis participating in a supervised exercise and patient education program in primary care in Denmark, stratified by affected joint (knee or hip)

Analgesic class	30-day interval relative to index date Number of DDDs per 1000 population																										
	Pre-intervention period intervals												Intervention period intervals			Post-intervention period intervals											
	-12	-11	-10	-9	-8	-7	-6	-5	-4	-3	-2	-1	0	1	2	3	4	5	6	7	8	9	10	11	12	13	14
Knee<br>*n = 26 462*																											
Paracetamol	3623	3717	3836	3721	3909	4123	4242	4311	4756	5102	5679	6286	5474	5335	5171	5487	5356	5246	5344	5672	5480	5409	5622	5495	5516	5515	5533
NSAIDs	2341	2365	2415	2398	2524	2696	2663	2780	3139	3341	3772	3987	3002	2967	2767	2948	2710	2776	2673	2692	2671	2644	2788	2503	2532	2476	2544
Opioids	1013	1055	958	987	1016	1057	1061	1029	1130	1154	1204	1180	1110	1079	1139	1221	1225	1237	1140	1251	1163	1153	1192	1175	1126	1116	1117
Gabapentinoids	402	340	396	386	384	397	403	370	403	377	398	421	404	422	413	406	444	425	447	477	462	446	438	461	414	453	517
SNRIs	734	832	783	790	789	706	741	735	733	790	748	789	718	790	770	742	848	668	817	816	733	748	854	832	722	844	818
TCAs	256	228	231	181	270	217	230	224	230	246	228	231	203	217	233	221	239	256	206	234	240	213	239	234	201	215	216
Hip<br>*n = 9075*																											
Paracetamol	3291	3581	3869	3620	3798	4221	4115	4561	4728	5260	6393	7382	6226	6417	6466	6378	6552	6817	6248	6551	6352	6542	6136	6089	6216	6234	6278
NSAIDs	1980	1996	1914	2050	1937	2226	2224	2494	2505	2797	3523	4176	2909	2975	3131	2881	2856	2839	2640	2571	2543	2523	2321	2441	2388	2023	2431
Opioids	914	950	935	882	940	962	958	932	1079	1021	1258	1258	1265	1255	1225	1305	1294	1284	1291	1136	1197	1171	1139	1149	1145	1130	1090
Gabapentinoids	280	361	313	325	332	342	373	302	336	358	335	387	387	364	410	400	408	402	438	482	394	454	420	443	460	466	502
SNRIs	749	743	745	706	734	745	788	721	736	749	736	744	774	750	617	735	974	685	670	935	758	638	966	861	807	788	824
TCAs	146	163	192	109	130	130	122	135	192	145	128	165	235	173	157	167	131	157	168	224	160	88	127	167	184	206	174

DDD, defined daily dose
NSAIDs, non-steroidal anti-inflammatory drugs
SNRIs, serotonin-norepinephrine reuptake inhibitors
TCAs, tricyclic antidepressants
30-day interval ‘0’ corresponds to the first 30-days of the intervention.

Table S7. Analgesic class-specific number of DDDs among 35 549 individuals with knee or hip osteoarthritis participating in a supervised exercise and patient education program in primary care and 675 286 individuals from a random matched general population sample in Denmark

Analgesic class	30-day interval relative to index date<br>Number of DDDs per 1000 population																										
	Pre-intervention period intervals												Intervention period intervals			Post-intervention period intervals											
	**-12**	**-11**	**-10**	**-9**	**-8**	**-7**	**-6**	**-5**	**-4**	**-3**	**-2**	**-1**	**0**	**1**	**2**	**3**	**4**	**5**	**6**	**7**	**8**	**9**	**10**	**11**	**12**	**13**	**14**
Study population n = 35 549																											
Paracetamol	3541	3686	3846	3703	3882	4148	4214	4375	4751	5144	5865	6567	5671	5612	5502	5721	5660	5648	5575	5900	5705	5701	5753	5649	5698	5698	5728
NSAIDs	2251	2273	2288	2312	2390	2579	2551	2709	2978	3202	3718	4040	2978	2970	2862	2944	2748	2793	2669	2665	2640	2616	2671	2492	2496	2360	2516
Opioids	987	1029	955	962	997	1034	1035	1006	1116	1121	1219	1202	1151	1124	1161	1244	1244	1249	1180	1223	1172	1159	1180	1169	1132	1120	1112
Gabapentinoids	370	345	374	371	371	383	395	353	387	372	381	412	400	407	412	404	435	420	446	478	444	449	433	457	426	456	514
SNRIs	737	813	773	768	779	716	753	735	733	779	749	777	732	784	731	740	880	672	784	847	744	719	883	840	744	834	820
TCAs	228	212	221	163	234	195	202	201	220	220	202	214	212	206	213	208	211	230	196	232	219	181	210	217	197	213	206
General population sample n = 675 286																											
Paracetamol	2882	2943	2952	2954	2963	3080	3027	3061	3102	3130	3117	3117	3278	3228	3210	3332	3307	3292	3338	3462	3383	3389	3493	3491	3512	3553	3653
NSAIDs	1340	1358	1328	1291	1316	1342	1309	1290	1292	1298	1270	1244	1301	1258	1243	1248	1226	1235	1201	1247	1224	1173	1217	1185	1175	1176	1189
Opioids	1175	1187	1188	1167	1160	1208	1170	1169	1185	1169	1167	1141	1193	1172	1140	1183	1160	1149	1150	1168	1140	1134	1169	1138	1148	1137	1172
Gabapentinoids	337	345	350	343	348	362	350	358	369	364	367	360	381	383	385	393	398	392	396	412	401	409	425	417	426	431	444
SNRIs	689	705	704	706	693	702	694	708	725	723	685	713	736	727	707	749	727	708	722	752	725	718	752	740	757	742	773
TCAs	207	218	211	208	215	217	213	202	212	212	209	209	209	215	204	212	207	202	209	211	208	203	211	203	202	205	215

DDD, defined daily dose
NSAIDs, non-steroidal anti-inflammatory drugs
SNRIs, serotonin-norepinephrine reuptake inhibitors
TCAs, tricyclic antidepressants
30-day interval ‘0’ corresponds to the first 30-days of the intervention.
Individuals from the general population sample were matched on year of birth, sex, municipality of residence, and being alive at the time of the index date.

Table S8. Analgesic class-specific number of DDDs among 35 549 individuals with knee or hip osteoarthritis participating in a supervised exercise and patient education program in primary care in Denmark, stratified by calendar year

**Calendar year / Analgesic class**	**30-day interval relative to index date Number of DDDs per 1000 population**																									
	**Pre-intervention period intervals**												**Intervention period intervals**			**Post-intervention period intervals**											
	**-12**	**-11**	**-10**	**-9**	**-8**	**-7**	**-6**	**-5**	**-4**	**-3**	**-2**	**-1**	**0**	**1**	**2**	**3**	**4**	**5**	**6**	**7**	**8**	**9**	**10**	**11**	**12**	**13**	**14**
2013-2015 n = 8988																											
Paracetamol	3037	3264	3497	3461	3531	3667	4164	4116	4461	4675	5569	6085	5182	5373	5200	5621	5299	5398	5306	5746	5349	5633	5483	5471	5601	5618	5392
NSAIDs	2403	2663	2578	2615	2666	3011	3088	3089	3526	3569	4137	4419	3311	3373	3013	3445	3082	3068	2908	2761	2783	2862	2982	2626	2791	2887	2771
Opioids	1053	1005	1021	965	1047	1190	1089	1077	1175	1189	1302	1192	1293	1231	1254	1340	1421	1264	1315	1332	1332	1338	1348	1303	1314	1374	1379
Gabapentinoids	326	276	324	290	317	306	325	252	336	323	310	316	346	355	311	327	390	318	386	366	368	385	343	349	378	359	415
SNRIs	753	685	562	807	680	632	610	577	692	657	675	636	665	648	765	630	704	483	688	864	680	507	810	624	824	657	839
TCAs	233	193	141	157	268	161	201	220	192	221	212	236	176	219	233	185	250	239	233	214	231	199	197	225	183	179	200
2016 n = 8697																											
Paracetamol	3966	4198	4051	3973	4191	4404	4251	4710	5233	5552	6113	6907	5897	5733	5510	6046	5957	5703	5615	6003	5966	5796	5932	5660	5657	5929	5913
NSAIDs	2296	2669	2616	2391	2509	2908	2721	3005	3288	3574	3937	4203	3121	3112	3095	3113	2945	3069	2796	2748	2903	2686	2746	2693	2507	2446	2767
Opioids	1090	1223	1023	1115	1142	1206	1145	1209	1293	1385	1417	1477	1336	1246	1381	1499	1505	1596	1444	1526	1375	1397	1368	1415	1279	1264	1286
Gabapentinoids	380	391	342	429	376	451	405	390	391	358	391	425	392	408	433	424	387	394	423	541	453	506	396	421	422	418	582
SNRIs	687	864	836	621	837	786	824	776	737	908	696	759	894	743	684	727	1064	737	705	765	813	784	841	895	799	856	959
TCAs	274	272	233	210	226	246	223	259	191	275	192	228	251	225	200	213	206	272	179	222	227	178	227	173	238	170	258
2017 n = 9042																											
Paracetamol	3590	3677	3904	3596	3965	4208	4124	4225	4603	5177	5718	6551	5668	5697	5532	5475	5680	5671	5527	5929	5662	5572	5674	5610	5706	5639	5798
NSAIDs	2249	1939	2111	2102	2282	2451	2272	2420	2713	2959	3508	4024	2905	2720	2738	2722	2566	2692	2547	2698	2445	2526	2504	2324	2329	2085	2290
Opioids	919	964	928	912	968	871	1030	916	1019	980	1082	1181	1069	997	1038	1151	1074	1169	965	1092	1010	964	1088	1005	937	1009	881
Gabapentinoids	406	353	400	355	385	370	391	390	347	404	387	427	369	405	416	420	461	458	478	425	443	385	411	485	392	466	446
SNRIs	703	737	853	697	830	698	699	736	761	792	777	867	770	859	671	931	957	608	902	892	679	671	980	945	610	878	747
TCAs	220	209	268	183	247	214	192	222	279	195	233	203	216	224	268	219	222	231	246	282	255	161	276	240	211	254	228
2018 n = 8329																											
Paracetamol	3583	3615	3944	3796	3846	4329	4325	4466	4719	5184	6085	6744	5964	5650	5786	5758	5717	5834	5873	5928	5860	5815	5942	5869	5834	5608	5817
NSAIDs	2042	1807	1827	2134	2086	1911	2102	2307	2354	2683	3268	3483	2554	2660	2592	2472	2381	2321	2412	2442	2426	2376	2440	2320	2350	2004	2226
Opioids	884	922	843	851	825	863	870	815	975	927	1072	947	894	1022	966	977	967	959	991	931	966	930	904	947	995	820	895
Gabapentinoids	370	361	434	416	406	408	464	380	480	406	441	486	500	465	494	450	505	513	502	590	518	526	594	579	521	588	623
SNRIs	810	980	847	958	767	752	890	862	743	761	855	850	595	890	808	666	791	878	839	864	810	932	897	897	744	951	731
TCAs	183	171	244	98	192	155	193	98	218	188	170	186	204	152	146	214	163	177	118	206	161	186	135	231	153	249	132

DDD, defined daily dose
NSAIDs, non-steroidal anti-inflammatory drugs
SNRIs, serotonin-norepinephrine reuptake inhibitors
TCAs, tricyclic antidepressants
30-day interval ‘0’ corresponds to the first 30-days of the intervention.
2013-2015 were collapsed due to low number of study participants.

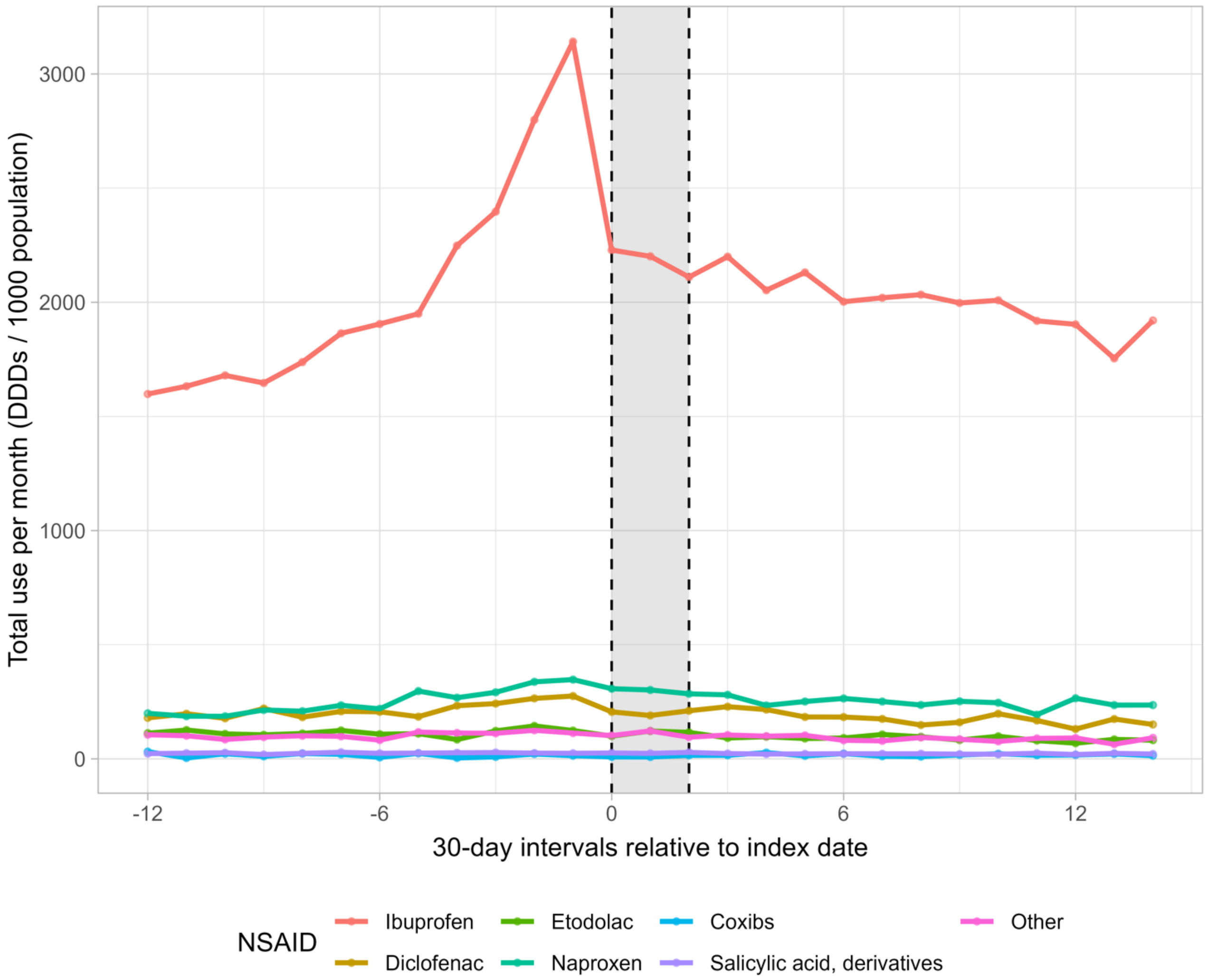

Figure S2. Total use of non-steroidal anti-inflammatory drugs (NSAIDs) per 1000 population and 30-days among 35 549 patients with knee or hip osteoarthritis before, during, and after an exercise therapy and patient education program in primary care in Denmark. The grey area reflects the intervention period. Interval 0 corresponds to the first month of the intervention. DDD is defined daily doses. NSAIDs is non-steroidal anti-inflammatory drugs.

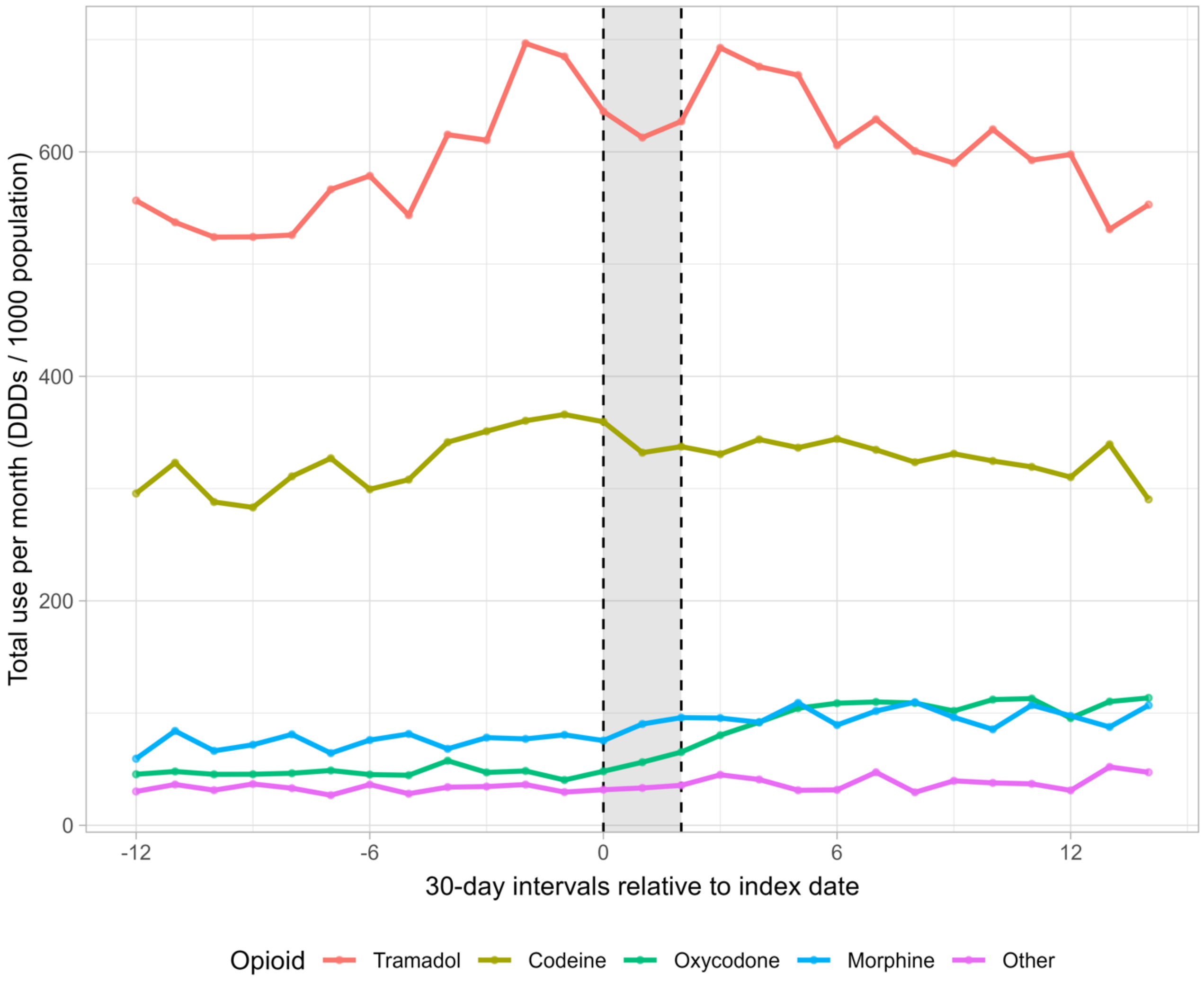

Figure S3. Total use of opioids per 1000 population and 30-days among 35 549 patients with knee or hip osteoarthritis before, during, and after an exercise therapy and patient education program in primary care in Denmark. The grey area reflects the intervention period. Interval 0 corresponds to the first month of the intervention. DDD is defined daily doses.

LORENZ CURVES

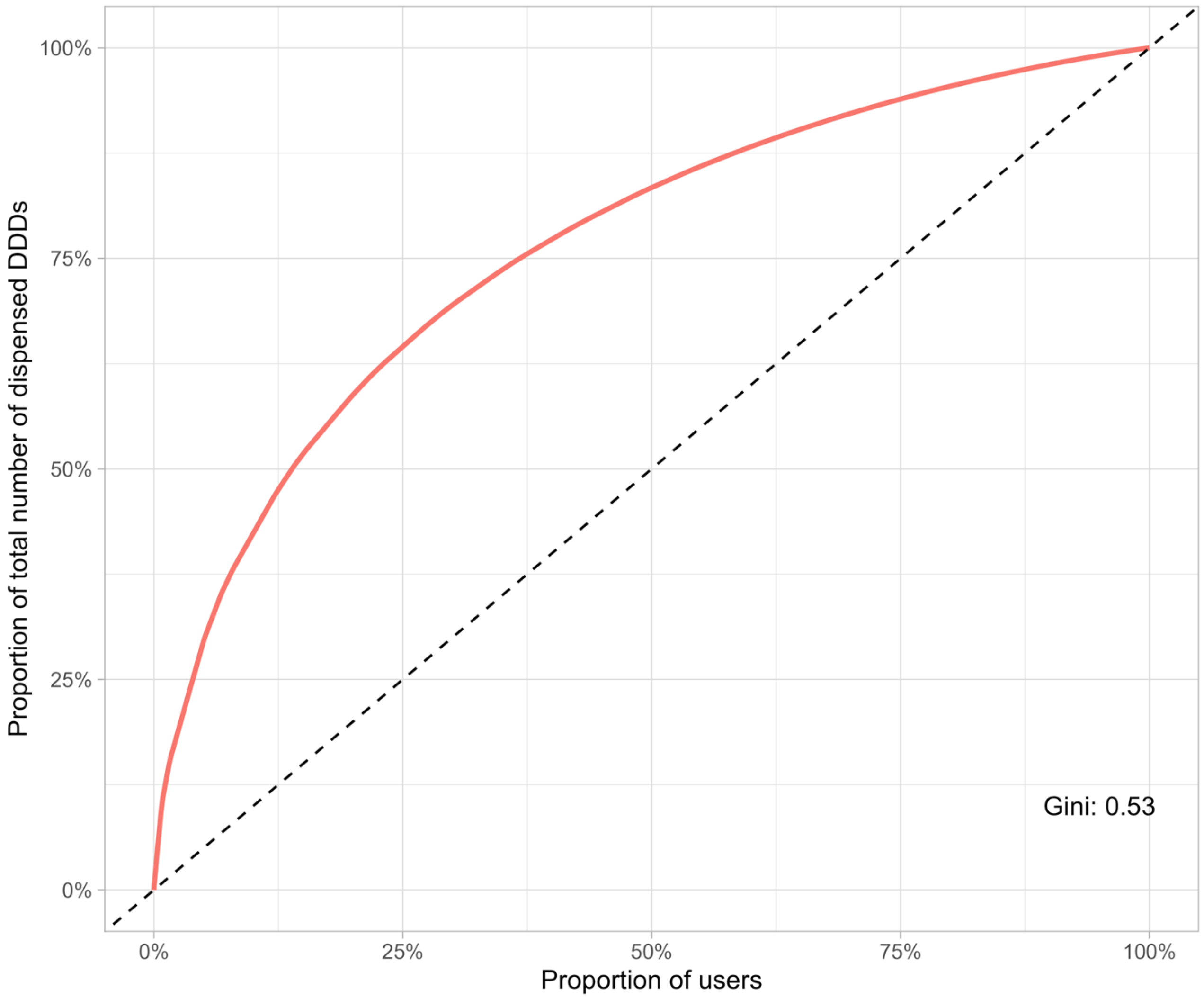

Figure S4. Lorenz curve illustrating the distribution of paracetamol use during the study period among 23 887 patients with knee or hip osteoarthritis participating in an exercise therapy and patient education program in primary care in Denmark. Only patients who dispensed a paracetamol prescription during the study period are in included. The Gini coefficient reflects the skewness of the Lorenz curve, where 0 reflects no skewness and 1 reflects maximal skewness. DDD is defined daily doses.

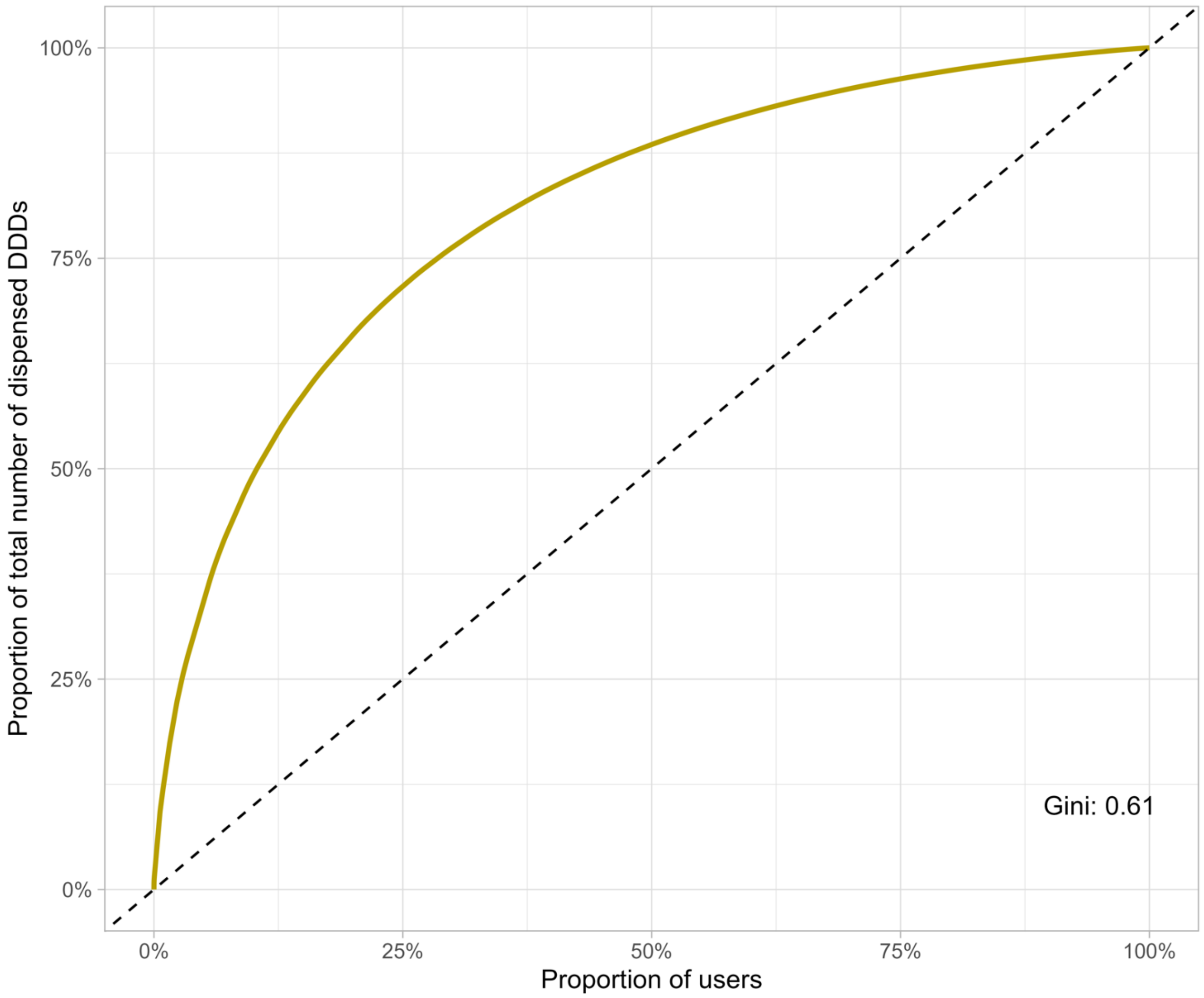

Figure S5. Lorenz curve illustrating the distribution of non-steroidal anti-inflammatory drug (NSAID) use during the study period among 19 572 patients with knee or hip osteoarthritis participating in an exercise therapy and patient education program in primary care in Denmark. Only patients who dispensed an NSAID prescription during the study period are in included. The Gini coefficient reflects the skewness of the Lorenz curve, where 0 reflects no skewness and 1 reflects maximal skewness. DDD is defined daily doses.

TOTAL DDDs DISPENSED DURING STUDY PERIOD

Table S9. Total number of DDDs dispensed during the study period among 35 549 patients with knee or hip osteoarthritis participating in an exercise therapy and patient education program, overall and by analgesic class

Overall / Analgesic class	Total DDDs (%)
Overall	9 973 707 (100)
Paracetamol	4 939 430 (50)
NSAIDs	2 620 275 (26)
Opioids	1 076 555 (11)
Gabapentinoids	394 465 (4)
SNRIs	741 743 (7)
TCAs	201 238 (2)

DDDs, defined daily doses
NSAIDs, non-steroidal anti-inflammatory drugs
SNRIs, serotonin–norepinephrine reuptake inhibitors
TCAs, tricyclic antidepressants
